# Genetic Alterations of *SMYD4* in Solid Tumors Using Integrative Multi-Platform Analysis

**DOI:** 10.3390/ijms25116097

**Published:** 2024-05-31

**Authors:** Brunna Letícia Olivera Santana, Mariana Braccialli de Loyola, Ana Cristina Moura Gualberto, Fabio Pittella-Silva

**Affiliations:** Laboratory of Molecular Pathology of Cancer, Faculty of Healthy Sciences, University of Brasília, Federal District, Brasília 70910-900, Brazil; brunna.los@hotmail.com (B.L.O.S.); marianabraccialli@outlook.com (M.B.d.L.); ana.gualberto@unb.br (A.C.M.G.)

**Keywords:** SMYD family, *SMYD4*, solid tumors, in silico analysis, protein methyltransferase, epigenetics

## Abstract

SMYD4 is a member of the SMYD family that has lysine methyltransferase function. Little is known about the roles of *SMYD4* in cancer. The aim of this study is to investigate genetic alterations in the *SMYD4* gene across the most prevalent solid tumors and determine its potential as a biomarker. We performed an integrative multi-platform analysis of the most common mutations, copy number alterations (CNAs), and mRNA expression levels of the *SMYD* family genes using cohorts available at the Cancer Genome Atlas (TCGA), cBioPortal, and the Catalogue of Somatic Mutations in Cancer (COSMIC). *SMYD* genes displayed a lower frequency of mutations across the studied tumors, with none of the *SMYD4* mutations detected demonstrating sufficient discriminatory power to serve as a biomarker. In terms of CNAs, *SMYD4* consistently exhibited heterozygous loss and downregulation across all tumors evaluated. Moreover, *SMYD4* showed low expression in tumor samples compared to normal samples, except for stomach adenocarcinoma. *SMYD4* demonstrated a frequent negative correlation with other members of the *SMYD* family and a positive correlation between CNAs and mRNA expression. Additionally, patients with low *SMYD4* expression in STAD and LUAD tumors exhibited significantly poorer overall survival. *SMYD4* demonstrated its role as a tumor suppressor in the majority of tumors evaluated. The consistent downregulation of *SMYD4*, coupled with its association with cancer progression, underscores its potential usefulness as a biomarker.

## 1. Introduction

Protein methyltransferases (PMTs) are a class of epigenetic modifiers essential for regulating a wide range of biological processes. Their misregulation can contribute to the development of various physiopathological conditions, including cancers. PMTs participate in crucial epigenetic processes by catalyzing the transfer of methyl groups to lysine or arginine residues. Molecular alterations in PMTs due to copy number alterations (CNA) or aberrant mRNA expression can lead to a series of dysfunctional events affecting several physiological conditions. The dysregulation of methylation processes has been linked to various pathological conditions, including cancer development, progression, and increased tumor aggressiveness, as well as metastatic events [1,2].

The SMYD (SET and MYND domain-containing proteins) family is a subgroup of protein methyltransferases that consists of five members (SMYD1-5). These proteins contain the SET domain, responsible for lysine methylation. The SET domain is divided into two segments by the MYND domain (Myeloid, Nervy, and DEAF-1), which is a unique characteristic of the SMYD family that facilitates protein-protein interactions [3]. While *SMYD4* has been less studied in carcinogenesis compared to other members of the *SMYD* family, it has been identified as a potential tumor suppressor due to its observed downregulation in breast cancer [4,5,6]. High expression of *SMYD4* has been implicated in the signaling pathways of cancer stem cells (CSCs), contributing to tumor cell transformation [7]. However, a comprehensive understanding of the molecular alterations in *SMYD4* within the context of a specific cancer remains largely unexplored.

According to the World Health Organization, the four most prevalent tumors among males in 2022, excluding non-melanoma skin cancer, were lung cancer (15.3%), prostate cancer (14.2%), colorectal cancer (10.4%), and stomach cancer (6.1%). In females, the leading types were breast cancer (23.8%), lung cancer (9.4%), colorectal cancer (8.9%), and cervix cancer (6.8%) [8]. Despite the heterogeneity of these cancer types, classical molecular signatures, such as mutations or alterations in key cell cycle regulators, like TP53, are often described [9]. Whether these cancer types also share common alterations in epigenetic genes is still unknown.

In this study, we conducted an integrative multi-platform analysis using publicly available databases to investigate the prevalent molecular alterations in the genes of the *SMYD* family, specifically in *SMYD4,* across eight of the most common tumor types. We analyzed data from breast invasive carcinoma (BRCA), prostate adenocarcinoma (PRAD), colorectal carcinoma (CRC), and stomach adenocarcinoma (STAD), as well as the most common subtypes of lung cancer, namely lung adenocarcinoma (LUAD) and lung squamous cell carcinoma (LUSC). Additionally, we examined uterine corpus endometrial carcinoma (UCEC) and uterine carcinosarcoma (UCS) cohorts from the Cancer Genome Atlas (TCGA) and cBioPortal. We aimed to uncover the potential prognostic values associated with these alterations.

## 2. Results

### 2.1. Mutation Profile of SMYD Genes

**Figure 1:**
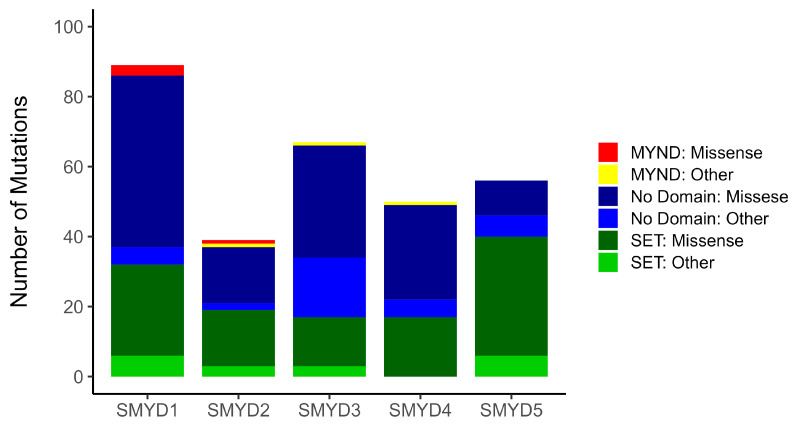
Total somatic mutations separated by conserved domains and non-conserved regions. Missense mutations in the SET domain are represented in dark green, and other mutations are noted in light green. Missense mutations in the MYND domain are represented in red, and other mutations are noted in yellow. Missense mutations in non-specific regions are represented in dark blue, and other mutations are noted in light blue.

We initially analyzed mutations in each *SMYD* family gene across the eight tumor types under examination. We used cohorts available at the cBio Cancer Genomics portal as well as at the Catalogue of Somatic Mutations in Cancer (COSMIC). The mutations in *SMYD* genes were examined in a total of 9696 patients encompassing all cancer types included in the analysis. Missense mutations were individually analyzed, as they were the most prevalent among the identified mutations, while other non-missense mutation types, such as frameshift, insertions, or deletions, were grouped and analyzed collectively. A total of 240 missense and 64 non-missense mutations were identified across the *SMYD* genes. The majority of missense mutations were located in non-conserved regions (129), followed by the SET domain (107) and the MYND domain (4). Specifically, *SMYD4* exhibited a total of 50 mutations, with 32 located in non-conserved regions, 17 in the SET domain and 1 in the MYND domain (Figure 1).

It was observed that the mutation frequencies within the *SMYD* family were relatively low, with certain genes exhibiting mutation frequencies higher than 2%. Specifically, *SMYD5* exhibited a mutation frequency of 2.3% in colorectal cancer, while *SMYD1* demonstrated a mutation frequency of 2.3% in both lung adenocarcinoma and lung squamous cell carcinoma. In endometrial carcinoma, the mutation frequencies were 3.3% for *SMYD1*, 2.1% for *SMYD2*, 2.1% for *SMYD3*, 2.1% for *SMYD4*, and 2.4% for *SMYD5*. Moreover, in uterine carcinosarcoma, the mutation frequencies were 5.1% for *SMYD1* and 2.5% for *SMYD4* (Table 1). These findings indicate that uterine tumors exhibited the highest mutation frequencies among the *SMYD* genes within the tumors evaluated, with particular emphasis on the mutation frequency of *SMYD1* in uterine carcinosarcoma.

However, no specific mutations across the *SMYD* genes were found to be frequently recurrent among patients with the same tumor type or within different tumors.

### 2.2. Copy Number Alterations in the SMYD Family Genes

Copy number alteration (CNA) refers to changes in DNA copy numbers occurring at specific locations in the genome. These alterations can lead to the activation of oncogenes or the suppression of tumor suppressor genes. Understanding the functionality of this mechanism is crucial for advancing the development of potential therapeutic and diagnostic markers [10]. We obtained CNA data for the *SMYD* genes through cBioPortal and analyzed these data using the GISTIC algorithm across all evaluated tumors [11].

**Figure 2:**
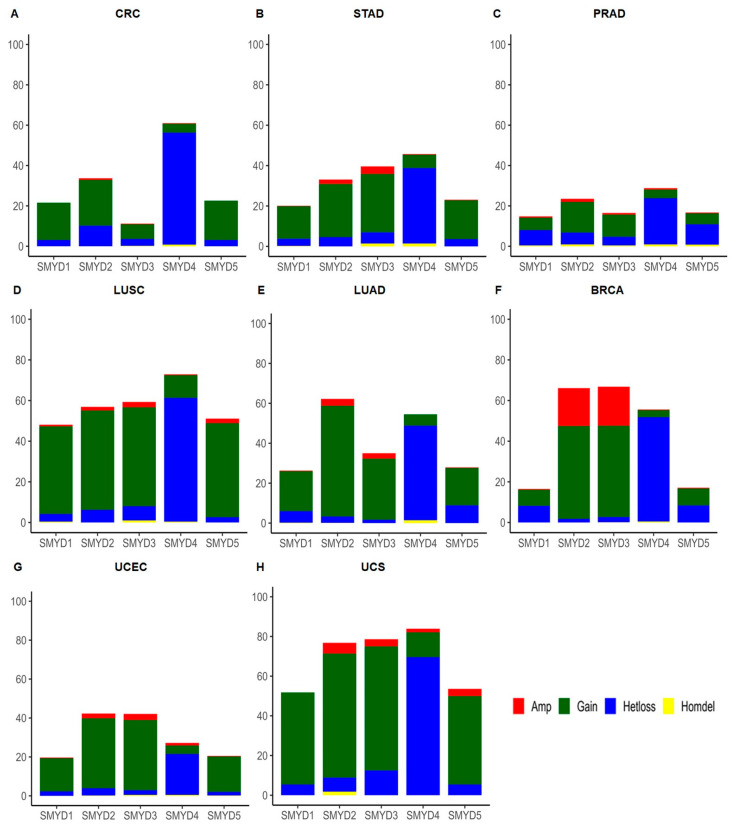
Frequency of copy number alterations in *SMYD* family genes. (**A**) Colorectal adenocarcinoma: n = 616 for *SMYD1, 2, 4,* and *5*; n = 1943 for *SMYD3*. (**B**) Stomach adenocarcinoma: n = 589. (**C**) Prostate adenocarcinoma: n = 2848 for *SMYD1, 2,* and *5*; n = 3890 for *SMYD3*; n = 1835 for *SMYD4*. (**D**) Lung squamous cell carcinoma: n = 501. (**E**) Lung adenocarcinoma: n = 1109 for *SMYD1, 2,* and *4*; n = 1973 for *SMYD3*; n = 1158 for *SMYD5*. (**F**) Invasive breast cancer: n = 3469. (**G**) Endometrial cancer: n = 620. (**H**) Uterine carcinosarcoma: n = 56. (Amp—high level amplification; Gain—low-level gain; Hetloss—heterozygous deletion; Homdel—homozygous deletion).

*SMYD4* exhibited a uniform alteration pattern, marked by a high incidence of heterozygous loss across all examined tumor types. These included colorectal adenocarcinoma (55.5%), stomach adenocarcinoma (37.4%), prostate adenocarcinoma (22.9%), lung squamous cell carcinoma (60.9%), lung adenocarcinoma (47.4%), breast carcinoma (51.3%), endometrial carcinoma (21%), and uterine carcinosarcoma (69.6%) (Figure 2A–H). Importantly, six out of eight tumors had heterozygous loss as the predominant CNA with a frequency of more than 30%. No other gene in the *SMYD* family exhibited a similar, consistently high level of heterozygous loss across the different tumor types.

In the case of *SMYD1*, the majority of alterations were observed as low-level gains in colorectal adenocarcinoma (18.5%), stomach adenocarcinoma (16.1%), lung squamous cell carcinoma (43.1%), lung adenocarcinoma (20%), endometrial carcinoma (17.1%), and uterine carcinosarcoma (46.4%). Prostate adenocarcinoma (7.6%) and breast carcinoma (8.2%) exhibited heterozygous loss as the most frequent alteration, although low-level gains exhibited frequencies of 6.2% and 7.9%, respectively (Figure 2A–H).

In all the tumors evaluated, low-level gains emerged as the most common alteration observed in *SMYD2* and *SMYD3*. Notably, in the case of breast carcinoma, the frequency of high-level amplification accounted for 18.6% and 19.2% of the alterations in *SMYD2* and *SMYD3*, respectively (Figure 2A–H). Finally, low-level gain was the most frequent alteration in *SMYD5* in colorectal adenocarcinoma (19.5%), stomach adenocarcinoma (19.2%), squamous cell lung cancer (46.3%), lung adenocarcinoma (18.7%), endometrial carcinoma (18.2%), and uterine carcinosarcoma (44.6%) tumors. Prostate adenocarcinoma (10.1%) was characterized by heterozygous loss. Breast carcinoma had similar distributions of low-level gain and heterozygous loss, with frequencies of 8.3% and 8.4%, respectively (Figure 2A–H).

### 2.3. mRNA Expression Profile

**Figure 3:**
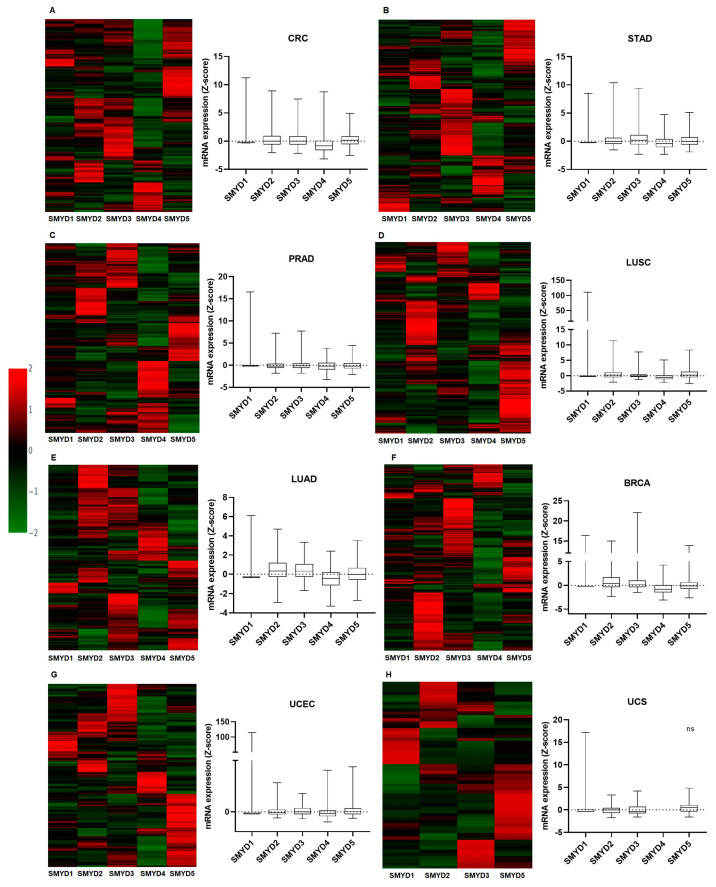
mRNA expression of *SMYD* family genes in solid tumors. (**A**) Colorectal cancer (n = 382). (**B**) Stomach adenocarcinoma (n = 415). (**C**) Prostate adenocarcinoma (*SMYD1*, *4,* and *5*: n = 498; *SMYD2*: n = 486; *SMYD3*: n = 493). (**D**) Lung squamous cell cancer (n = 501). (**E**) Lung adenocarcinoma (n = 169). (**F**) Invasive breast cancer (1100). (**G**) Uterine corpus endometrial carcinoma (n = 177). (**H**) Uterine carcinosarcoma (n = 57). Low expression was defined as a value below the 50th percentile, and high expression was defined as a value above the 50th percentile. *SMYD4* expression data for UCS were not available.

mRNA expression was assessed using Z-scores relative to diploid sample data obtained for each cancer type from cBioPortal. Heatmaps and boxplots were used to assess the expression levels of the *SMYD* genes in all eight cancer types. In line with previous results observed for CNA, *SMYD4* also exhibited a uniform expression pattern in all tumors evaluated. In all tumors cohorts, *SMYD4* followed a pattern of downregulation (Figure 3A–H). This result corroborates the CNA findings, which indicated a high frequency of heterozygous loss (Figure 2A–H). These observations are consistent with previous studies suggesting a potential tumor suppressor function for *SMYD4* [4,5,6]. Among other *SMYD* family genes, *SMYD4* was the only one with such a consistent downregulation pattern across the different tumor types.

*SMYD1* was downregulated in CRC, BRCA, UCEC, and UCS tumors, while its expression was upregulated in STAD, PRAD, LUSC, and LUAD. A similar variation in mRNA expression levels was observed for *SMYD2*. While its expression was increased in the majority of CRC, STAD, LUSC, LUAD, and BRCA tumors, it was downregulated in patients with PRAD, UCEC and UCS tumors. *SMYD*3 showed upregulated expression levels in CRC, STAD, LUAD, BRCA, and UCEC, while it was downregulated in the majority of PRAD and UCS patients. *SMYD*3 remained at basal levels in LUSC (Figure 3A–H). *SMYD5* was upregulated in the majority of CRC, STAD, LUSC, LUAD, UCEC, and UCS tumors. In contrast, it was downregulated in PRAD and BRCA patients (Figure 3A–H). 

With the exception of *SMYD4*, which exhibited a consistent downregulated pattern, all the other members of the *SMYD* family showed a distinct pattern of expression depending on the tumor.

### 2.4. Correlation Analysis of SMYD4 Expression with Other SMYD Genes

**Figure 4:**
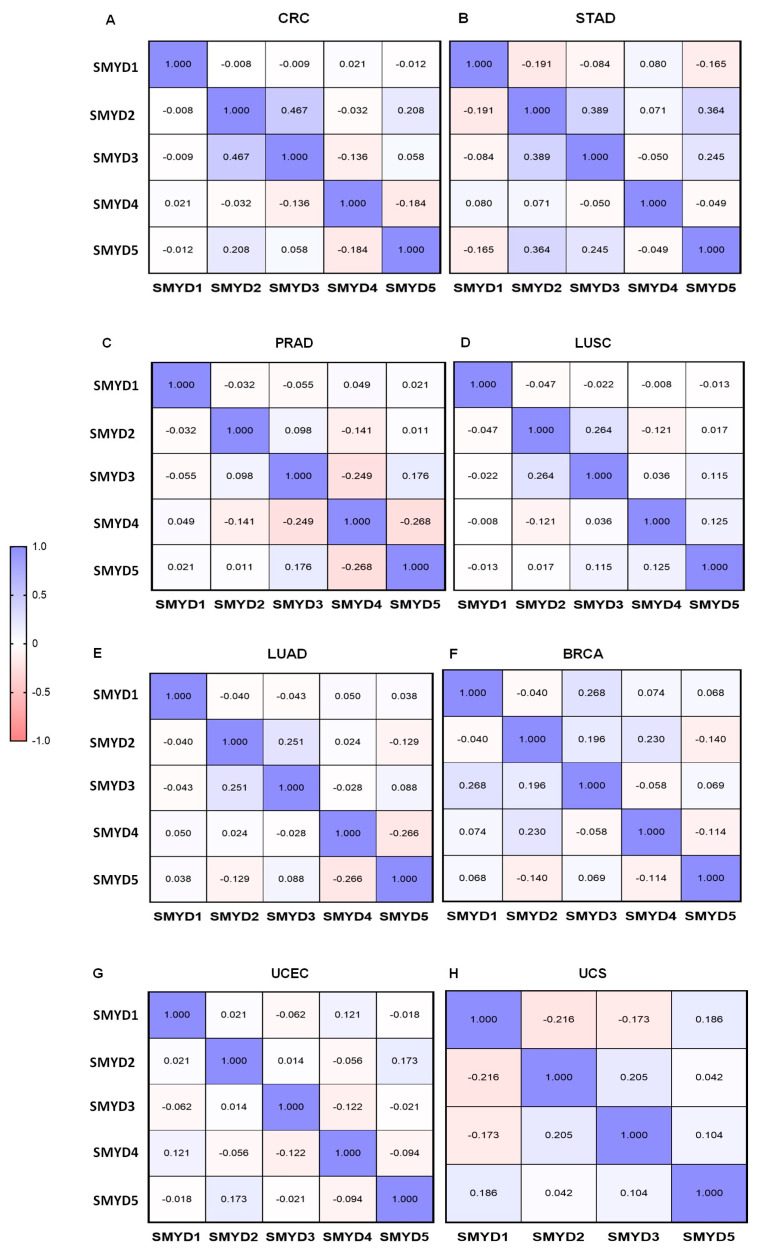
Correlation of mRNA expression of *SMYD* family genes in solid tumors. (**A**) Colorectal cancer (n = 382). (**B**) Stomach adenocarcinoma (n = 415). (**C**) Prostate adenocarcinoma (*SMYD1, 4,* and *5*: n = 498; *SMYD2*: n = 486; *SMYD3*: n = 493). (**D**) Lung squamous cell cancer (n = 501). (**E**) Lung adenocarcinoma (n = 169). (**F**) Invasive breast cancer (1100). (**G**) Uterine corpus endometrial carcinoma (n = 177). (**H**) Uterine carcinosarcoma (n = 57). Pearson correlation test was used for comparison of expression levels among the five genes in each tumor evaluated (*p* = 0.05). Red represents a negative correlation (r values close to −1). Blue represents a positive correlation (r values close to +1). White represents independent variables with values close to 0.

The correlation of *SMYD4* expression with other *SMYD* family genes was assessed using Pearson correlation analysis. The analysis revealed weak negative correlations between *SMYD4* and *SMYD3* (r = −0.136, *p* = 0.008) and between *SMYD4* and *SMYD5* (r = −0.184, *p* = 3.015 × 10^−4^) in CRC (Figure 4A). In STAD, there was no significant correlation between *SMYD4* and the other *SMYDs* (Figure 4B). In PRAD, Pearson’s correlation also showed poor negative correlations between *SMYD4* and *SMYD2* (r = −0.141, *p* = 0.002), *SMYD4* and *SMYD3* (r = −0.249, *p* = 1.761 × 10^−8^), and *SMYD4* and *SMYD5* (r = −0.268, *p* = 1.309 × 10^−9^) (Figure 4C).

In LUSC, a slightly positive correlation was observed between *SMYD4* and *SMYD5* (r = 0.125, *p* = 0.005). A poor negative correlation was observed between *SMYD2* and *SMYD4* (r = −0.121, *p* = 0.007) (Figure 4D). In LUAD, a weak negative correlation was also observed between *SMYD4* and *SMYD5* (r = −0.266, *p* = 4.666 × 10^−4^) (Figure 4E).

There was also a minimal positive correlation between *SMYD1* and *SMYD4* (r = 0.074, *p* = 6.045 × 10^−5^) and a low negative correlation between *SMYD3* and *SMYD4* (r = −0.058, *p* = 4.967 × 10^−5^) and between *SMYD4* and *SMYD5* (r= −0.114, *p* = 0.002) in BRCA (Figure 4F). In UCEC, a poor positive correlation was observed between *SMYD2* and *SMYD4* (r = −0.056, *p* = 0.002) (Figure 4G). In UCS, there was no significant correlation between *SMYD4* and the other members of the *SMYD* family (Figure 4H).

### 2.5. Comparison of SMYD4 Expression between Normal Samples and Tumor Samples at Different Tumor Stages

**Figure 5:**
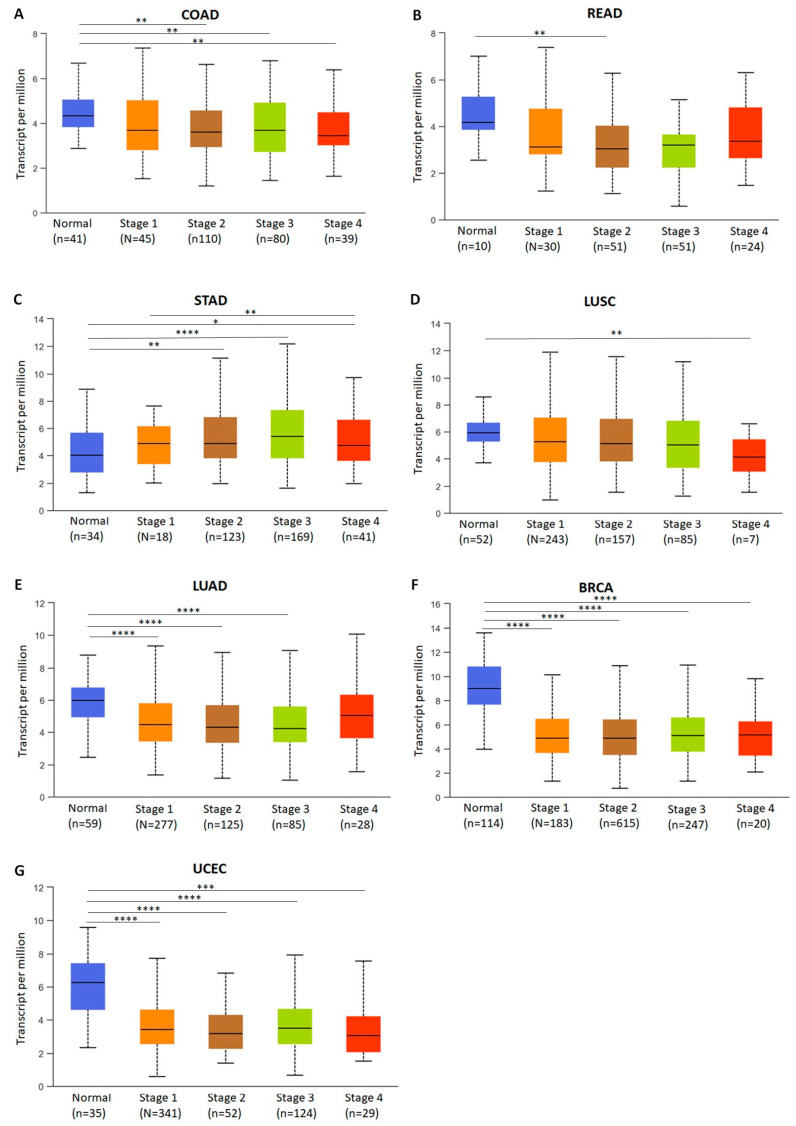
*SMYD4* expression between normal and tumor stages samples. (**A**) Colon adenocarcinoma (COAD). (**B**) Rectum adenocarcinoma (READ). (**C**) Stomach adenocarcinoma (STAD). (**D**) Lung squamous cell carcinoma (LUSC). (**E**) Lung adenocarcinoma (LUAD). (**F**) Invasive breast cancer (BRCA). (**G**) Uterine corpus endometrial carcinoma (UCEC). No data were available for normal samples comparison to UCS. (* *p* < 0.05; ** *p* < 0.01; *** *p* < 0.001; **** *p* < 0.0001 with statistical significance).

*SMYD4* exhibited a negative regulation pattern based on mRNA analysis in all the evaluated tumors. To assess the expression of *SMYD4* in normal samples and tumor samples at different disease stages, we profiled its mRNA expression using the UALCAN database. In this analysis, colon and rectal tumors were evaluated separately, and it was observed that *SMYD4* expression in colon adenocarcinoma was significantly lower in stage 2, 3, and 4 tumors (*p* = 1.04 × 10^−3^, 5.55 × 10^−3^, and 1.21 × 10^−2^, respectively) when compared to normal samples. In rectal adenocarcinoma, significantly low expression of *SMYD4* was only observed in stage 2 tumor samples (*p* = 1.86 × 10^−2^) when compared to normal sample (Figure 5A,B).

The expression of *SMYD4* in STAD was noteworthy for the fact that it differed from the other tumors in that its expression was significantly higher in stages 2, 3, and 4 when compared to the normal sample (*p* = 3.83 × 10^−3^, 2.28 × 10^−5^, and 3.37 × 10^−2^, respectively). Stage 3 had a statistically significant higher expression of *SMYD4* when compared to stage 1 (*p* = 1.94 × 10^−2^) (Figure 5C). In LUSC, *SMYD4* expression was significantly lower in stage 4 compared to normal samples (*p* = 1.55 × 10^−3^) (Figure 5D). In LUAD, patients with stage 1, 2, and 3 tumors presented low expression when compared to normal samples (*p* = 1.19 × 10^−6^, *p* = 7.68 × 10^−8^, and *p* = 3.51 × 10^−5^, respectively) (Figure 5E). In BRCA, *SMYD4* expression was also significantly lower in all tumor stages in comparison with normal samples (*p* < 1 × 10^−12^, *p* = 1.11 × 10^−16^, *p* < 1 × 10^−12^, and *p* = 1.96 × 10^−12^ for stages 1, 2, 3, and 4, respectively) (Figure 5F). In UCEC, low expression of *SMYD4* was observed in stages 1, 2, 3, and 4 (*p* = 3.26 × 10^−11^, 7.71 × 10^−10^, 1.30 × 10^−9^, and 7.80 × 10^−4^, respectively) when comparing them with normal samples (Figure 5G).

### 2.6. Correlation Analyses between SMYD4 CNAs and mRNA Expression

A correlation analysis was conducted to ascertain the relationship between *SMYD4* copy number alterations (CNAs) and mRNA expression across various tumor types. These included colorectal cancer (CRC), gastric adenocarcinoma (STAD), lung squamous cell carcinoma (LUSC), breast invasive carcinoma (BRCA), and uterine corpus endometrial carcinoma (UCEC). We found a statistically significant positive correlation for all tumors evaluated in this study, indicating that CNAs influenced mRNA expression in these tumors. Specifically, CRC, STAD, LUSC, and BRCA had a Pearson correlation coefficient greater than 0.500. LUSC and UCEC had Spearman correlation coefficient greater than 0.500 (Table 2).

### 2.7. Overall Patient Survival Based on SMYD4 Expression

**Figure 6:**
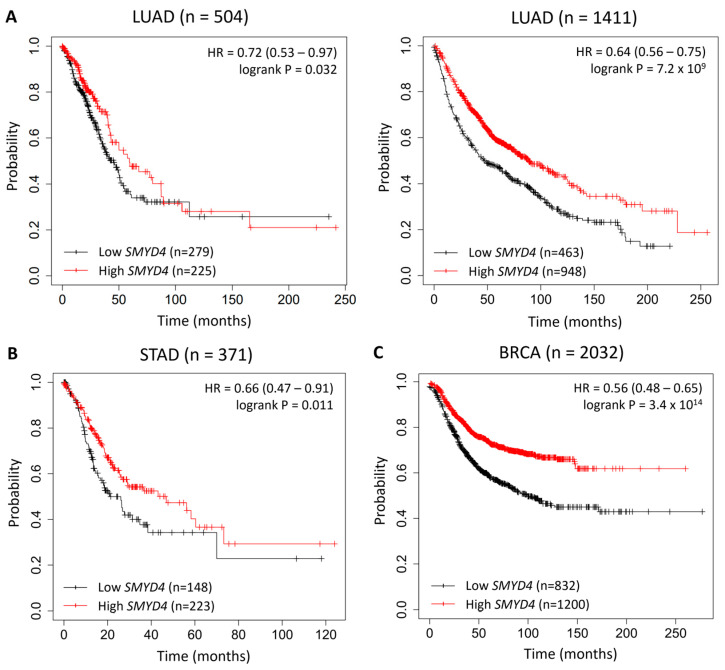
Overall survival (OS) or relapse-free survival (RFS) analysis based on *SMYD4* expression levels (Kaplan-Meier plotter). (**A**) Two distinct cohorts of lung adenocarcinoma patients (LUAD) were analyzed. The left panel shows the OS in a cohort of 371 patients based on RNA-seq data. The right panel shows the OS of a cohort of 1411 patients based on microarray data. (**B**) OS in a cohort of 371 stomach adenocarcinoma (STAD) patients. (**C**) RFS in a cohort of 2032 breast cancer patients (n = 2031) was analyzed based on microarray data. Differences in OS or RFS were analyzed with the log-rank test.

To assess whether there is a correlation between the overall survival (OS) of patients and the expression level of *SMYD4*, we analyzed its mRNA expression using both RNA-Seq data and microarray data (Affymetrix ID 229175_at) available from the Kaplan–Meier plotter database. For each cancer type evaluated, we dichotomized patients based on *SMYD4* expression into two groups with high or low expression as previously described [12]. We found that low expression of *SMYD4* was significantly linked to a reduced OS in LUAD patients. In a cohort of 504 LUAD patients, those with lower expression of *SMYD4* had a median overall survival of 45.2 months compared with an overall survival of 59.2 months of patients with higher expression (HR = 0.72, 95% CI = 0.53–0.97; *p* = 0.032) (Figure 6A). This difference became even more pronounced in a separate cohort of 1411 LUAD patients, where *SMYD4* expression was analyzed using microarrays. Patients exhibiting lower *SMYD4* expression had a median overall survival of 47 months. In contrast, patients with higher *SMYD4* expression demonstrated a median overall survival of 88.7 months (HR = 0.64, 95% CI = 0.56–0.75; *p* = 7.2 × 10^−9^) (Figure 6A). Similarly, in a cohort of 371 STAD patients, those with higher expression had a median overall survival of 46.9 months, whereas those with lower *SMYD4* expression had a median overall survival of 21.1 months (HR = 0.66, 95% CI = 0.47–0.91; *p* = 0.011) (Figure 6B). 

When we analyzed available cohorts of BRCA patients, although *SMYD4* expression did not significantly affected OS, it was linked with a worse relapse-free survival (RFS). Patients who had lower expression of *SMYD4* demonstrated a median RFS of 28 months compared with the group that had higher expression (median RFS of 59 months, HR = 0.56 95% CI = 0.48–0.65; *p* = 3.4 × 10^−14^) (Figure 6C).

## 3. Discussion

The availability of vast amounts of cancer genomic data in public database repositories has made in silico analysis an indispensable tool for exploring cancer-related vulnerabilities. Analyzing the most predominant genetic alterations in *SMYD* genes has revealed important distinctions among each member, their relation with cancer progression, and their usefulness as prognostic tools. 

In the context of mutations, the missense variant emerges as the most frequently observed mutation across *SMYD* genes. The conserved SET domain, in particular, harbors a total of 107 missense mutations within the *SMYD* gene family. However, none of these mutations demonstrate a clear predominance, making it challenging to determine their potential impact on the functional dynamics of the *SMYD* family.

*SMYD4* emerged as a point of interest in our analysis, as all the evaluated tumors displayed a high frequency of heterozygous loss. This observation is consistent with the mRNA findings across all analyzed tumor types, which all showed a trend toward downregulation. This trend may be due to the fact that *SMYD4* is located on 17p13.3, a region known to undergo heterozygous loss in various solid tumors and leukemias [4,13,14,15,16]. Although not a rule, copy number alterations can affect gene expression [17]. The correlation analysis between CNAs and mRNA expression in all evaluated tumors showed that both alterations in *SMYD4* are correlated. 

Intriguingly, Xiao et al. proposed that *SMYD4* is accountable for di- and tri-methylation at H3K4 in zebrafish [18]. H3K4me3 is an epigenetic marker recognized for preserving the activity of tumor suppressor genes in normal cells [19]. Thus, if the function of *SMYD4* observed in zebrafish is conserved and mirrors its role in humans, SMYD4 could potentially activate tumor suppressors through H3K4me3. Therefore, a loss in *SMYD4* function might lead to a corresponding loss of function in other tumor suppressors.

To date, very few studies have examined the role of *SMYD4* in cancer. Our analysis confirmed that *SMYD4* was downregulated in the majority of BRCA patients examined. Although there was no association with OS, downregulation of *SMYD4* significantly affected RFS. Importantly, BRCA patients also presented a significant correlation between CNAs and mRNA expression. This observation supports the findings that propose a tumor suppressor role for *SMYD4* in the development of breast cancer, at least partially, by inhibiting platelet-derived growth factor receptor α polypeptide (Pdgrf-α) [4]. Its downregulation also helps in the process of transforming normal mammary cells into tumor cells [5]. In addition, a study by Zhang et al. also found that *SMYD4* was downregulated in PRAD tumor tissues, which is also consistent with the findings of our study [20]. We also demonstrated that *SMYD4* consistently displayed a pattern of downregulation across a variety of tumor types, implying that its role as a tumor suppressor might be a universal characteristic in diverse oncological contexts.

Importantly, a comparative analysis of *SMYD4* expression between samples from normal tissues and samples from tumors at different stages revealed that *SMYD4* expression is significantly decreased in cancer samples, regardless of the tumor stage. Despite no association with tumor stage, *SMYD4* expression remains significantly reduced during cancer progression. This was observed in almost all cancer types analyzed, including colon and rectal cancers, breast cancer, uterine corpus endometrial carcinoma, and lung squamous cell carcinoma. For patients with lung adenocarcinoma, a decrease in *SMYD4* expression also resulted in a lower overall survival rate. This suggests that *SMYD4* expression could be explored as a valuable biomarker for predicting a more severe prognosis in this cancer type.

Interestingly, in the examined data from stomach adenocarcinoma, specifically those from stage 2, 3, and 4 tumors, *SMYD4* exhibited higher expression levels compared to normal samples in most patients. However, Kaplan–Meier analysis of 371 patients revealed that lower expression of *SMYD4* was associated with poorer overall survival, indicating a more unfavorable outcome among those patients with reduced *SMYD4* expression. One possible explanation for this intriguing alteration in *SMYD4* expression in STAD may be that higher *SMYD4* potentiates the expression of the transcription factor Nanog in cancer stem cells [7]. Previous studies have demonstrated a correlation between elevated Nanog expression in gastric tumors and increased tumor aggressiveness, as it enhances cell proliferation, migration and invasion [21,22]. A recent study also reports that *SMYD4* is upregulated in hepatocellular carcinoma, forming a positive feedback loop with the arginine methyltransferase PRMT5 [23]. As the role of *SMYD4* in cancer becomes more evident, further research is needed to better comprehend its mechanism of action in these tumor types.

The correlation analysis revealed noteworthy findings regarding changes in the expression pattern between *SMYD4* and other members of the *SMYD* family. In general, *SMYD4* negatively weak correlates with other *SMYD* genes. For instance, *SMYD3* is known to be upregulated in colorectal cancers [24,25,26,27]. In patients with high expression of *SMYD3*, *SMYD4* is consistently downregulated, exhibiting a clear negative correlation. Interestingly, a similar correlation was found in CRC between *SMYD4* and *SMYD5*, which is a much less studied gene. This negative correlation was also observed in PRAD, where patients with a high expression of *SMYD2*, *SMYD3* [26,28], or *SMYD5* showed lower expression of *SMYD4*. This opposing expression pattern was also observed in LUSC and BRCA, where *SMYD4* is downregulated while *SMYD5* is upregulated. In BRCA, there was a small negative correlation between *SMYD4* and *SMYD3*, which is expected since *SMYD3* upregulation is already known to be associated with breast cancer proliferation [26,27,29,30]. Despite sharing similar conserved domains, the contrasting expression patterns of these genes in cancer may suggest divergent roles.

In conclusion, our study highlights *SMYD4* as a tumor suppressor gene across various solid tumors. Our comprehensive analysis of genetic alterations within the *SMYD4* gene revealed consistent heterozygous loss and downregulation across all tumors evaluated. Furthermore, *SMYD4* exhibited frequent negative correlations with other members of the *SMYD* family, suggesting distinct functional roles. Importantly, the consistent downregulation of *SMYD4* expression and its association with poor overall survival underscores its likely role in tumorigenesis and highlights its potential as a valuable biomarker. Further research into the mechanisms underlying *SMYD4*’s tumor-suppressive functions is warranted to fully elucidate its clinical potential.

## 4. Materials and Methods

### 4.1. Gene Database Analysis

Mutation data were obtained from the cBio Cancer Genomics Portal (www.cbioportal.org) and the Catalogue Of Somatic Mutations In *Cancer* (COSMIC) “www.cancer.sanger.ac.uk/cosmic (accessed on 31 October 2022)”. Data on copy number alterations and mRNA expression were obtained from the cBio Cancer Genomics Portal “www.cbioportal.org (accessed on 31 July 2022)” and the Cancer Genome Atlas (TCGA) database “www.cancergenome.nih.gov (accessed on 31 June 2022). The frequency of copy number alterations (CNAs) was generated using the algorithm GISTIC (Genomic Identification of Significant Targets in Cancer), and the copy number of each gene in each tumor evaluated was determined, where −2 indicates a homozygous loss, −1 is heterozygous loss, 1 is low-level gain, and 2 indicates amplification. For mRNA expression analysis, data from patient samples relative to diploid samples were used. The value is presented as a standard deviation of the mean expression (Z-score) [20,31]. The UALCAN database “http://ualcan.path.uab.edu (accessed on 31 August 2023)”, which contains data obtained from TCGA [32], was used to evaluate the relative expression of *SMYD4* between normal and tumor stage samples in order to identify whether *SMYD4* could serve as a potential biomarker in the tumors evaluated in this study. Graphical representations were generated using GraphPad Prism 8 and Rsudio 2023.06.0 software.

### 4.2. Heatmap

The z-score data related to the mRNA expression of *SMYDs* in the evaluated cancer types were visualized using heatmap plots generated through the Flaski data analysis and visualization tool “https://flaski.age.mpg.de (accessed on 30 September 2023)”. The data were scaled numerically within a range of ±2. The Ward linkage method with Euclidean distance was employed for clustering. The data were sorted into row clusters.

### 4.3. Survival Curve

The Kaplan–Meier plotter database “http://kmplot.com/analysis/ (accessed on 30 April 2023)” was used to explore the prognostic significance of alterations in the five *SMYD* genes. Survival analyses were conducted to determine the correlation between *SMYD* gene alterations and tumor prognosis. For mRNA expression analysis, the mRNA pan cancer option was selected, and patients were divided based on “high expression” and “low expression” levels as previously described [12]. OS for LUAD and PFS for BRCA were also assessed using data from microarray assays. *p* value < 0.05 was considered statistically significant.

### 4.4. Statistics

Statistical analyses were performed using GraphPad Prism 8. The mRNA correlations between *SMYD* family genes were analyzed using Pearson correlation test, with *p* < 0.05 serving as the definition of a statistically significant difference.

## Figures and Tables

**Table 1:** Mutation frequency in each *SMYD* family member among the eight tumor types.

Tumor	*SMYD1*	*SMYD2*	*SMYD3*	*SMYD4*	*SMYD5*
CRC	1.4%	0.9%	1.0%	0.5%	2.3%
STAD	1.2%	0.9%	1.9%	0,8%	1.1%
BRCA	0.3%	0.2%	0.4%	0.4%	0.3%
PRAD	0.2%	0.1%	0.2%	0.1%	0.1%
Lung Cancer					
LUAD	2.3%	0.8%	0.7%	0.4%	0.5%
LUSC	2.3%	0.8%	1.2%	0.8%	0.4%
Uterine Cancer					
UCEC	3.3%	2.1%	2.1%	2.1%	2.4%
UCS	5.1%	0.0%	0.0%	2.5%	1.3%

**Table 2:** Correlation analyses of *SMYD4* mRNA and CNA.

Tumors	Pearson	*p* Value	Spearman	*p* Value	Patients (n)
CRC	0.568	<0.0001	0.337	<0.0001	255
STAD	0.593	<0.0001	0.465	<0.0001	190
PRAD	0.468	<0.0001	0.296	0.0015	98
LUSC	0.615	<0.0001	0.500	<0.0001	362
LUAD	0.488	<0.0001	0.354	0.0003	89
BRCA	0.532	<0.0001	0.472	<0.0001	695
UCEC	0.483	<0.0001	0.538	<0.0001	74

## Data Availability

Data is contained within the article.
